# Melatonin Immunoreactivity in Malignant Small Intestinal Neuroendocrine Tumours

**DOI:** 10.1371/journal.pone.0164354

**Published:** 2016-10-13

**Authors:** Fanny Söderquist, Eva Tiensuu Janson, Annica J. Rasmusson, Abir Ali, Mats Stridsberg, Janet L. Cunningham

**Affiliations:** 1 Department of Neuroscience, Psychiatry, Uppsala University, Uppsala, Sweden; 2 Department of Medical Sciences, Section of Endocrine Oncology, Uppsala University, Uppsala, Sweden; 3 Department of Medical Sciences, Clinical Pharmacology and Osteoporosis, Uppsala University, Uppsala, Sweden; 4 Department of Medical Sciences, Biochemical Endocrinology, Uppsala University, Uppsala, Sweden; University of Alabama at Birmingham, UNITED STATES

## Abstract

**Background/Aims:**

Small intestinal neuroendocrine tumours (SI-NETs) are derived from enterochromaffin cells. After demonstrating melatonin in enterochromaffin cells, we hypothesized that SI-NETs may express and secrete melatonin, which may have an impact on clinical factors and treatment response.

**Methods:**

Tumour tissue from 26 patients with SI-NETs, representing paired sections of primary tumour and metastasis, were immunohistochemically stained for melatonin and its receptors, MT_1_ and MT_2_. Plasma melatonin and immunoreactivity (IR) for melatonin, MT_1_ and MT_2_ in tumour cells were compared to other tumour markers and clinical parameters. Melatonin was measured at two time points in fasting morning plasma from 43 patients with SI-NETs.

**Results:**

Melatonin IR was found in all SI-NETS. Melatonin IR intensity in primary tumours correlated inversely to proliferation index (p = 0.022) and patients reported less diarrhoea when melatonin IR was high (p = 0.012). MT_1_ IR was low or absent in tumours. MT_2_ expression was medium to high in primary tumours and generally reduced in metastases (p = 0.007). Plasma-melatonin ranged from 4.5 to 220.0 pg/L. Higher levels were associated with nausea at both time points (p = 0.027 and p = 0.006) and flush at the second sampling. In cases with disease stabilization or remission (n = 34), circulating melatonin levels were reduced in the second sample (p = 0.038).

**Conclusion:**

Immunoreactive melatonin is present in SI-NETs. Circulating levels of melatonin in patients with SI-NETs are reduced after treatment. Our results are congruent with recent understanding of melatonin’s endocrine and paracrine functions and SI-NETs may provide a model for further studies of melatonin function.

## Introduction

Small intestinal neuroendocrine tumours (SI-NETs) are tumours derived from enterochromaffin (EC) cells. These tumours are generally well differentiated with low proliferation rate and survival expectancy longer than for other NETs [1]. SI-NETs are generally sporadic although familial cases have been reported [2]. EC-cells and consequently SI-NETs are known to produce and secrete a number of bioactive agents including serotonin and tachykinins such as substance P and neurokinin A [3]. These hormones contribute to the “carcinoid syndrome”; a set of symptoms that include diarrhoea, cutaneous flush, bronchial constriction and carcinoid heart disease, which generally appear when the tumour has metastasized to the liver. Most patients present with disseminated disease and treatment is often focused on reduction of tumour burden, slowing tumour progression and ameliorating symptoms with primarily somatostatin analogues and/or interferon-alpha [4–6].

Melatonin, first isolated by Lerner *et al*, is an indoleamine, two enzymatic steps from serotonin, produced by the pineal gland and promotes sleep [7]. A replicated finding is lower peripheral evening or night-time melatonin levels in depressed patients compared to healthy controls [8]. Melatonin receptor agonists are now used in psychiatric care for treatment of sleeping disorders, depression and anxiety and may have potential for treating other disorders [9].

Melatonin also acts in an autocrine, paracrine and endocrine manner mediating a wide spectrum of regulatory metabolic functions, which includes glucose homeostasis and vascular effects [10]. Studies by Slominski *et al* have shown pathways for melatonin synthesis in human skin and melatonin may play a significant role in preserving skin barrier and in protection against UV-induced skin damage [11–13]. Melatonin can reduce toxicity and adverse side effects of cancer treatment and has also been shown to have independent oncostatic effects in various types of cancer [14].

We have recently described the expression of melatonin and its receptors, MT_1_ and MT_2_ in the normal human gastrointestinal (GI) tract and pancreas [15]. Expression of melatonin as well as MT_2_ was found in EC-cells, but not MT_1_. Melatonin in the GI-tract differs in circadian rhythm from pineal produced melatonin and varies in relation to fasting and food intake. Levels of melatonin in the gastrointestinal tissues greatly exceed serum levels [16]. In the GI-tract of animals, melatonin appears to act as a physiological antagonist of serotonin in regulating motility and it protects the mucosa by increasing bicarbonate secretion [17, 18]. Melatonin expression in SI-NETs is yet to be investigated.

## Objectives

This study aimed to examine the immunohistochemical expression of melatonin and its receptors, MT_1_ and MT_2,_ in SI-NETs in relation to proliferation index and local symptoms. Furthermore, we investigated levels of circulating melatonin in patients with SI-NETs in relation to symptoms and outcome measures.

## Materials and Methods

### Patient samples

Clinical data were collected retrospectively from electronic medical records for 52 patients with SI-NETs, diagnosed between 1976 and 2007 at the Laboratory of Pathology and Cytology and treated at the Department of Endocrine Oncology, Uppsala University Hospital in Sweden. Patients were included in plasma analyses based on availability of paired data from two sampling occasions (n = 43). Patients were included in immunohistochemical analyses based on availability of both primary and metastatic tissue, quality of tumour material and clinical data (n = 26). Seventeen patients were included in both parts of the study. The diagnosis of SI-NET was based on international recommendations for the classification of endocrine tumours. Plasma-chromogranin A (CgA), urinary 5-hydroxyindole acetic acid (U-5-HIAA), radiology and histopathological investigations were performed at diagnosis [19].

The data extracted from the medical records, from time of blood sampling or operation, included age at diagnosis, BMI, smoking history, diagnosis of diabetes, use of psychiatric drugs that potentially modify serotonin and/or melatonin levels (antidepressants, anti-anxiety drugs and medication for sleep disorders), U-5-HIAA and CgA. Smoking history was classified as current, past or no smoking history. Symptoms of diarrhoea, nausea and/or vomiting and flushing were also documented. Information of tumour grade was collected from the histopathological statement at the time of diagnosis for patients in plasma analyses. For patients included in the immunohistochemical analyses, Ki67 index was re-evaluated for each case on serial sections. The disease stage and clinical data were documented either at time of plasma sampling or time of operation for the respective patient groups.

For detailed patient characteristics see Table 1. All patients in the immunohistochemical analyses had disseminated disease at the time of surgery, for detailed tumour classification data see Table 2.

**Table 1:** Clinical characteristics for patients submitted to analyses of plasma and tissue.

	Tissue analyses (n = 26)	Plasma analyses (n = 43)
**Age (years)**	61 [42–86]	61 [26–79]
**Sex**		
female	13	23
male	13	20
**BMI (kg/m**^**2**^**)** ^a^	23.6 [14.9–31.8]	25.0 [14.9–41.9]
**Smoking history**		
no	15	26
past	9	12
current	2	5
**Diabetes**	3	5
**Psychiatric drugs**		
antidepressants	3	6
anti-anxiety drugs	2	5
sleeping pills	5	6
**WHO classification, stage**		
IIb	0	2
IIIa	0	0
IIIb	6	16
IV	20	25
**Tumour grade**^**b**^		
G1	21	31
G2	5	6
G3	0	0
**Metastases**		
Lymph node	26	41
Liver	20	25
Distant^c^	10	9
**Carcinoid syndrome**	21	37
**Treatment before analysis**		
Medical ^d^	14	7
Surgery	1	22
Untreated	12	17
**Survival (months)** ^e^	101 (79–122)	144 (122–166)

^a^ BMI = Body Mass Index; Missing data for 3 patients in plasma analyses and for 2 patients in tissue analyses.

^b^ G1 ki67 index <2%, G2 Ki67 index 3–20%, G3 Ki67 index >20%. Missing data for patients (n = 6).

^c^ Distant metastases: peritoneal carcinomatosis (n = 4), lung (n = 2), bone (n = 2), ovary (n = 1), breast (n = 1), pancreas (n = 1), orbita (n = 1).

^d^ Medical treatment includes interferon-alpha, somatostatin analogues or a combination of the two.

^e^ Mean (95% confidence interval), censored data for 2 patients in plasma analyses and for 5 patients in tissue analyses.

**Table 2:** Biomarkers, symptoms, treatment and response at plasma sampling 1 and 2 in 43 patients.

	Sampling 1	Sampling 2
**Melatonin** median [range]	26.0 [4.5–220.0]	23.0 [8.9–90.7]
**CgA** median [range]	8.4 [1.6–355.0]	5.7 [1.7–121.0]
**U-5-HIAA** median [range]	73.0 [10.0–1380.0]	36.5 [10.0–1130.0]
**Clinical symptoms**		
Diarrhoea	30	25
Nausea/vomiting	11	10
Flush	25	16
**Treatment**		
INF	4	38
SOM	5	18
INF+SOM	2	13
Surgery	22	16^a^
Untreated	17	0
**Treatment response**		
Stable disease		18
Regressive disease		16
Progressive disease		9

^a^ Only patients that underwent surgery between samplings are reported.

Abbreviations: CgA = chromogranin A, U-5-HIAA = urinary 5-hydroxyindoleacetic acid, INF = interferon-alpha, SOM = somatostatin analogues.

### Immunohistochemistry (IHC) and microscopy assessments

Tissue specimens were fixed in 4% buffered formalin for 1–2 days, dehydrated, and embedded in paraffin wax. Sections, 4 μm thick, were attached to positively charged glass superfrost slides (Menzel-Gläser, Braunschweig, Germany), deparaffinized in xylene and rehydrated using decreasing concentrations of ethanol to distilled water. Antigen retrieval was performed using pressure cooker treatment for 10 minutes in citrate buffer pH 6. For MT_1_, sections were incubated for 30 minutes in 0.1% H_2_O_2_ to quench endogenous peroxidase, washed in phosphate-buffered saline (PBS), and then incubated with normal horse serum diluted 1:5 for 30 minutes. (Vector Laboratories, Burlingame, CA, USA). The sections were then incubated overnight at 4°C with a polyclonal goat anti-human MT_1_ antibody (Anti-MTR-1A, (N-20), sc13179, Santa Cruz Biotechnology, Dallas, Texas, USA diluted 1:500). Sections were washed thrice in PBS and then incubated with biotinylated horse anti-goat antibody (BA-9500, Vector Laboratories Peterborough, United Kingdom). Thereafter, sections were incubated for 30 minutes with avidin-biotin–horseradish peroxidase (PK-6100, Vectastain Elite ABC kit, Vector Laboratories, Peterborough, United Kingdom). Rabbit anti-melatonin (0100–0203 AbD Serotec, Kidlington, UK, diluted 1:500), rabbit anti-melatonin receptor 1B (MT_2_) (ABIN122307, Antibodies-online GmbH, Aachen, Germany diluted 1:100), monoclonal mouse anti-serotonin (Clone 5HT-H209 DAKO Sweden AB, Stockholm, Sweden, diluted 1:100) and monoclonal mouse anti-human Ki67 (Clone MIB-1, DAKO, Glostrup, Denmark, diluted 1:100) antibodies were diluted in PBS with 1% bovine serum albumin (BSA). The DAKO EnVision™ systems for rabbit and for mouse (DAKO Sweden AB, Stockholm, Sweden) were used for antibody detection according to the manufacturer’s instructions. Diaminobenzidine (DAB) was used as chromogen. For visualization of nuclei, the sections were counterstained with Mayer’s haematoxylin.

**Fig 1:**
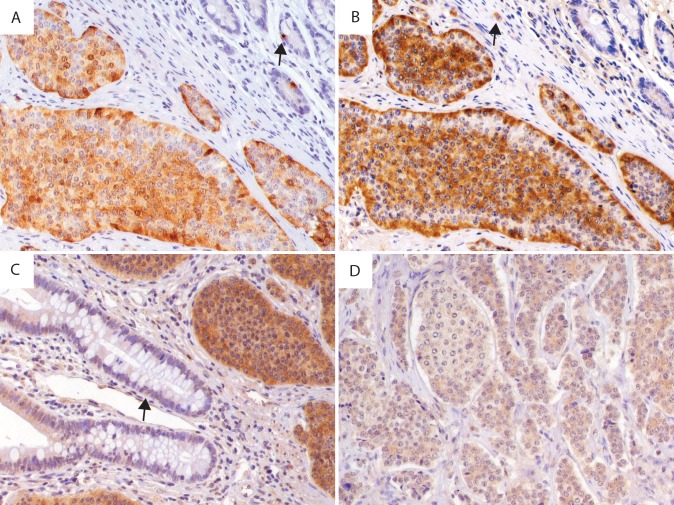
A–D. Microphotograph of SI-NETs illustrating expression of serotonin, melatonin and MT_2_. **A)** Serotonin in primary tumour, arrow indicating positive serotonin IR in EC-cell. **B)** Melatonin in the same tumour, arrow indicating strong positive melatonin IR in immune cell. **C)** Strong MT_2_ IR in primary tumour, arrow indicating positive MT_2_ IR in EC-cell**. D)** Weak MT_2_ IR in metastasis from the same patient as C. Magnification 200 times.

Tumour material consisted of serial sections of primary tumours (n = 26) and metastases (n = 26). All specimens were coded and examined microscopically by transmitted light using Zeiss Axiophot microscope and Axiovision software (version SE64, Rel. 4.9.1, Carl Zeiss AB, Stockholm, Sweden) by three different observers, performed blinded at different occasions using both low (100-200x) and high magnification (400x) and images were acquired with a colour camera with the same illumination and the colour balance corrected. All tumour sections were subjectively assessed regarding a positive or negative expression for melatonin, serotonin, MT_1_ and MT_2_ as well as the intensity of the staining when positive. Immunoreactivity (IR) on sections was classified according to a scoring system including negative (0), weak (1), moderate (2) or strong (3). Very high correlation was found between the evaluations of different observers, the intraclass correlation coefficient, Cronbach’s Alpha, was 0.955 indicating high reliability. In the few cases when the observers assigned different scores, slides were evaluated together and consensus was reached. To further ensure reliability in scoring, sections were analysed using Image J software. The DAB-brown staining was identified using the Color Threshold plugin with the colour space set to HSB and the hue set to range 0–70. Median signal intensity in two tumour areas for each section was evaluated by FIJI/Image J software [20]. Three groups based on intensity scores were defined and labelled weak, moderate and strong (see Fig 1C and 1D and S1 Fig). When comparing manual scoring with the computerized scoring, Cronbach’s Alpha was 0.934.

Positive control sections from tissues known to express MT_1_ and MT_2_ (skin, pancreas) were used for comparison. In tumour sections assessed as negative, staining was present in control cells (epithelium, immune cells).

### Antibody specificity tests

We have previously reported the antibody specificity tests and results [15]. In summary, control immunostaining included omission of the primary antisera and replacement of the primary antibody by non-immune serum at the same dilution as the primary antibody in question in the same diluent was performed for all antibodies. A neutralisation test was performed for melatonin antibodies by preincubation in saturated solutions of melatonin (0.1 mg/mL) or serotonin (0.1 mg/mL) in PBS with 1% BSA. Specificity tests using preincubation with corresponding peptides were also performed for the MT_1_ and MT_2_ antibodies using IHC. A 24-hour incubation of primary antiserum with the relevant peptide (sc-13179P, Santa Cruz Biotechnology, Dallas, Texas, USA and SP4391CP, Acris Antibodies GmbH, Herford, Germany, 10 nmol peptide per mL, diluted 1:100 antibody solution) was performed before application to the sections.

### Plasma analyses

Plasma samples were collected in the morning after an overnight fast at two occasions (sampling 1 and 2) and stored at -80°C. Treatment with surgery, interferon-alpha, somatostatin analogues or any combination of the three was documented for both time points. For three patients, there was no change in treatment between sampling 1 and 2. Response to treatment between samplings was classified as regression, stabilization or progression, according to radiological and/or biochemical response. Biochemical progression was defined as an increase in either CgA or U-5-HIIA by >25% and regression as a decrease by >50%. A specialist in radiology using computerized tomography and/or ultrasound evaluated radiological progression or regression of metastases. Patients with stabilization or tumour regression were compared to those with tumour progression in statistical analyses.

Plasma levels of melatonin were measured using competitive radioimmunoassay (Melatonin direct RIA, LDN, Norhorn, Germany), all samples were analysed at the same time using a kit from the same batch. Total coefficient of variation was less than 10%.

### Statistical analyses

Data was stored in an Access database and analysed using the statistical program package SPSS 21.0 (SPSS Inc., Chicago, IL, USA). Statistical analyses were conducted separately for the two groups of patients; those where histopathological specimens were available (n = 26) and those where plasma samples were collected (n = 43). The cohorts were not compared to each other. Patient characteristics were presented in descriptive statistical analyses. Survival was calculated from time of diagnosis to death. For patients still alive, time of diagnosis until present date was used to calculate survival. Five patients were lost to follow-up and they were censored in survival analyses.

For statistical analyses of differences between the intensity of IR in primary tumours and metastases and for change in biomarker response between samplings, Wilcoxon Signed Ranks Test was used. Spearman’s test was used for analyses of correlations between IR or plasma melatonin and clinical parameters. The Mann-Whitney U test was used to look for sex differences in circulating levels of melatonin in plasma. Survival analyses were performed using the Mantel-Cox log rank test.

Results are presented as median [range] unless otherwise stated. A p-value less than 0.05 was considered significant. Effect size for the Wilcoxon Signed Ranks test was calculated using the formula *r* = *Z*/(√*n*) [21].

### Ethics statement

The study was approved by the local ethics committee of the Uppsala University Hospital (Dnr: 2007/143 and 2007/143/1 GK 2012-09-14) and all included patients signed an informed consent.

## Results

### Antibody specificity tests

Preincubation of melatonin antibodies with saturated serotonin solution marginally blocked the melatonin immunostaining (see S2 Fig and S3 Fig). Only partial overlap between melatonin and serotonin is seen in co-localisation studies [15] and comparison of serial section, (see S4 Fig). Staining with melatonin, MT_1_ and MT_2_ antibodies were completely blocked by preincubation with the respective antigens (see S5 Fig and S6 Fig).

### Expression of melatonin IR in SI-NETs and correlation to clinical parameters

Melatonin IR was found in all primary tumours (n = 26) and metastases (n = 26), see Table 3. IR intensity in primary tumours was weak in 6 cases, medium in 11 cases and strong in 9 cases. In metastases, weak IR was seen in 10 cases, while 9 were medium and 7 showed strong IR. No significant difference in melatonin IR intensity between primary tumours and metastases was found (r = 0.29; p = 0.134). Immunohistochemical staining is illustrated in Fig 1.

**Table 3:** Expression of melatonin (Mel), receptors (MT_1_ and MT_2_) and serotonin (Ser) in primary tumours and metastases assessed by immunohistochemistry. Difference (p-value) in expression between primary tumours (n = 26) and metastases (n = 26).

Antibody	IR	Primary tumour (n = 26)	Metastasis (n = 26)	P value^a^
**Melatonin**	Positive IR	100%	100%	
	Weak	6	10	
	Medium	11	9	
	Strong	9	7	
				0.134
**MT**_**1**_	Positive IR	19%	23%	
	Negative	21	20	
	Weak	5	6	
				0.655
**MT**_**2**_	Positive IR	100%	92%	
	Negative	0	2	
	Weak	7	13	
	Medium	14	8	
	Strong	5	3	
				**0.007**
**Serotonin**	Positive IR	96%	100%	
	Negative	1	0	
	Weak	0	4	
	Medium	5	4	
	Strong	20	18	
				0.206

^a^ Wilcoxon Signed Ranks Test

IR = Immunoreactivity

A negative relationship was found between melatonin IR in primary tumours and the proliferation marker Ki67 (r = -0.446; p = 0.022). Melatonin IR in primary tumours as well as in metastases showed an inverse correlation to diarrhoea (r = -0.484; p = 0.012 and r = -0.398; p = 0.044, respectively) but for other symptoms reported, no significant correlations were found. There were no significant correlations between melatonin IR and sex, BMI, diabetes, use of psychiatric drugs or survival.

### Expression of MT_1_IR and MT_2_IR in SI-NETs and correlation to clinical parameters

MT_1_ IR was low or absent in the majority of sections studied. In primary tumours, 21 sections were negative and 5 sections showed weak IR. In metastases, 20 were negative and 6 sections were weakly positive (see Table 3). Positive MT_1_ IR was found in the brush border of the epithelium, which served as an internal positive control. No significant difference in MT_1_ IR between primary tumours and metastases was found (r = 0.088; p = 0,655). MT_1_ IR in primary tumours showed a significant positive correlation for the symptoms of diarrhoea (r = 0.418; p = 0.034), as well as for nausea and/or vomiting (r = 0.732; p = 0.00002). All sections positive for MT_1_ IR were from female patients and the sex difference was statistically significant for both primary tumours (r = -0.488; p = 0.011) and metastases (r = -0.548; p = 0.004). No significant correlation between MT_1_ IR and flush, BMI, diabetes, use of psychiatric drugs, Ki67 Index or survival was found.

MT_2_ IR was positive in all 26 sections from primary tumours, 7 sections were weakly stained, 14 medium and 5 sections showed strong IR intensity. In metastases, 2 sections were negative, 13 weakly stained, 8 medium and 3 strongly positive. Intensity of MT_2_ IR was generally lower in metastases than in primary tumours (r = 0.53; p = 0.007). Positive expression for MT_2_IR in tumour sections is illustrated in Fig 1. No significant association between MT_2_ IR and clinical parameters including sex, symptoms of flush, diarrhoea, or nausea and/or vomiting, BMI, use of psychiatric drugs, Ki67 Index or survival was found.

### Expression of serotonin IR in SI-NETs

All tumours with exception of one primary tumour expressed serotonin IR. The majority showed strong IR, in primary tumours (n = 20) and in metastases (n = 21). The remaining sections showed medium IR. No correlations between serotonin IR and melatonin IR, Ki67 Index, or carcinoid syndrome symptoms were found.

### Analyses of plasma samples from patients with SI-NETs

Median plasma melatonin level at the first sampling was 26.0 pg/L [4.5–220.0] and at the second sampling 23.0 pg/L [8.9–90.7], see Table 2. A wider range of values of circulating melatonin was seen at the first sampling occasion compared to the second. Age did not correlate with melatonin values at either occasion (r = -0.129, p = 0.409; r = 0.074, p = 0.636). In patients with disease stabilization or regression at the second sampling, melatonin levels were reduced at the second sampling (r = 0.356; p = 0.038) and the largest reductions were seen in patients with the highest levels of melatonin at the first sampling. The tumour markers CgA and U-5-HIAA were also reduced in these patients (r = 0.731; p = 0.00003 and r = 0.828; p = 0.000003).

A significant sex difference in levels of circulating melatonin was noted at both sampling occasions, with higher levels in women than in men (r = 0.46; p = 0.003 and r = 0.39; p = 0.010, respectively). There was no difference between men and women in treatment response. A positive relationship between plasma melatonin levels and symptoms of nausea and/or vomiting was found at both time points (r = 0.337, p = 0.027 and r = 0.413; p = 0.006) and at the second time point there was also a correlation to flush (r = 0.353; p = 0.020). There was no significant correlation at either of the sampling occasions between plasma levels of melatonin and use of psychiatric drugs, BMI, tumour grade or survival.

## Discussion

This is the first study to demonstrate expression of immunoreactive melatonin in SI-NETs. Ubiquitous expression of melatonin in SI-NETs agrees with earlier findings of melatonin IR and gene expression of the enzymes needed for the production of melatonin from serotonin, arylalkylamine N-acetyltransferase and acetylserotonin O-methyltransferase, in EC-cells [15]. The expression of immunoreactive melatonin in SI-NETs correlates to a lower proliferation index in this study, which is in agreement with the anti-neoplastic effects of melatonin described in many studies. For example, anti-proliferative effects were demonstrated for melatonin in murine colon cancer [22]. Melatonin induced caspase activity and apoptosis in human malignant lymphoid cell lines [23] and caused cell cycle arrest in HepG2 human hepatocarcinoma cells [24]. In neural and blood cells and other normal tissues, however, melatonin promotes cell survival by reducing apoptosis through decreased activity of caspase-9 and -3 [25–27]. It is possible that melatonin contributes to the paradoxical clinical phenotype of many SI-NETs where low proliferation and apoptosis rates but nonetheless malignant behaviour is described [28].

Previous studies have identified oncostatic effects of melatonin mediated by the MT_1_ receptor. For instance a recent study in colorectal adenocarcinoma showed reduced expression of MT_1_ when compared to adjacent normal tissue and it was speculated that MT_1_ may mediate anti-tumuorigenic effects [29]. MT_1_ IR was low or absent in both primary and metastatic tissue which is in line with a model where tumour cells are able to proliferate despite elevated levels of melatonin. Binding of melatonin to the MT_1_ receptor has been reported to up-regulate cyklin dependent kinase inhibitor gene (CDKN1B/p27) in prostate cancer [30]. Interestingly, the tumour suppressor p27 is highly expressed in most SI-NETs; however, mutations in the gene encoding p27, leading to dysregulation of the cell cycle, have been proposed as an underlying genomic mechanism in the pathogenesis of SI-NETs [31, 32].

Immunoreactivity for the MT_2_ receptor, on the other hand, was identified in all tumours studied; however, the intensity of MT_2_ IR was much lower in metastases than in primary tumours. MT_2_ intensity did not correlate to Ki67 Index. This loss of MT_2_ receptor expression could represent decreased sensitivity to anti-tumourigenic effects of melatonin and merits further investigation.

A wide range of values for morning plasma melatonin was found in patients with SI-NETs. Surprisingly these values were not correlated with age. Although melatonin is produced in different organs, the largest source of circulating melatonin is usually the pineal gland during nocturnal hours [33]. Melatonin production is drastically reduced with age and was expected to be lower in the study population where the median age is 61. After treatment (interferon-alpha, somatostatin, surgery alone or in combination) levels of melatonin, CgA and U-5HIAA were reduced in patients with tumour regression or stabilization, which indicates that, a reduction of tumour size and/or activity results in a lower amount of circulating melatonin. In relation to clinical parameters, high levels of circulating melatonin were significantly correlated to the symptoms of nausea and/or vomiting. A slower gastrointestinal transit time could hypothetically cause nausea, which is in agreement with the proposed actions of melatonin in regulation of gastrointestinal motility [16]. Related to this, the immunohistochemical expression of melatonin correlated inversely to the symptom of diarrhoea, indicating less trouble with diarrhoea when a high intensity of melatonin IR was found in the tumour. Melatonin is a small lipophilic compound that diffuses easily through cell membranes and quickly accesses different tissues allowing for both paracrine and endocrine actions. The functional effects of melatonin in the gastrointestinal tract in humans are still being explored and animal studies have shown ambiguous and dose-dependent results regarding the effect of melatonin on motility [17, 34]. Our findings support earlier claims for a future role for melatonin in the therapy for diarrhoeal diseases for example ulcerative colitis and irritable bowel syndrome [35]. Circulating levels of melatonin at the second sampling occasion also correlated to the symptom of flush. Flush in the carcinoid syndrome is primarily associated with the release of serotonin and tachykinins [36], but melatonin has also been shown to have vasoactive effects [37].

We found a sex difference, with higher circulating levels of melatonin in the females than in the males, which is in agreement with previous findings of analyses of melatonin in overnight urine [38]. Besides sex, a wide variety of factors could influence gastrointestinal melatonin e.g. age [39], antidepressant medication [40] and BMI [41]. We did not find associations to these factors. It is possible that other confounding factors, yet to be identified, also affect circulating levels of melatonin.

The data from medical records was collected retrospectively, which limits the possible analyses that can be performed. There was no correlation between levels of melatonin and the use of antidepressants, anti-anxiety drugs or medication for sleeping disorders. Low melatonin is associated with sleeping disorders and other psychiatric conditions [8]. Theoretically, therapy that reduced melatonin levels may increase psychiatric symptoms and the psychiatric effects of hypermelatoninism are unknown. A study specifically designed to capture psychiatric symptoms is necessary to further explore these issues.

## Conclusions

SI-NETs display immunoreactive melatonin and in patients with SI-NETs, levels of melatonin in plasma are reduced after treatment. In a subgroup of patients, circulating melatonin is associated with clinical symptoms with nausea and flush via endocrine or paracrine mechanisms. We found that patients with high tumour expression of immunoreactive melatonin, in addition to less diarrhoea, had lower tumour proliferation. Furthermore, the expression of the melatonin receptor MT_2_ was down regulated in metastases, which could indicate reduced sensitivity to melatonin signalling as important for tumour progression. These findings are congruent with recent understanding of melatonin’s biological functions in animal models and SI-NETs may provide a model for further studies of melatonin function.

## Supporting Information

S1 Fig Intensity scoring for melatonin immunohistochemistry. Representative examples of melatonin immunohistochemistry intensities of tumour tissue scored using both manual and computerised methods, weak (A), moderate (B) and strong (C).
(TIF)

S2 Fig Melatonin antibody neutralisation test. Melatonin immunohistochemistry of normal human rectal epithelium stained using rabbit-anti melatonin antibody 0100–0203 (A). Melatonin immunohistochemistry of a consecutive section where antibody was pre-incubated with 0.1 mg/mL melatonin to ensure antibody specificity (B).
(TIF)

S3 Fig Melatonin antibody cross-reactivity with serotonin. Melatonin immunohistochemistry of normal ileal epithelium stained using rabbit-anti melatonin antibody 0100–0203 (A). Melatonin immunohistochemistry of a consecutive section where antibody was pre-incubated with 0.1 mg/mL serotonin to ensure antibody specificity (B). Serotonin immunohistochemistry of consecutive section stained using anti-serotonin antibody 5HT-H209 (C). Arrowheads indicate enterochomaffin cells.
(TIF)

S4 Fig Different staining patterns for melatonin and serotonin on consecutive sections. Melatonin immunohistochemistry of small intestinal neuroendocrine tumour stained using rabbit-anti melatonin antibody 0100–0203 (A). Serotonin immunohistochemistry of a consecutive section stained using anti-serotonin antibody 5HT-H209 (B). Arrowheads indicate cluster of tumour cells.
(TIF)

S5 Fig Melatonin receptor 1 (MT1) antibody neutralisation test. Immunohistochemistry of normal ileal epithelium tissue stained using MT1 antibody sc13179 (A). Immunohistochemistry of a consecutive section where the antibody was pre-incubated with the corresponding peptide sc-13179P (B).
(TIF)

S6 Fig Melatonin receptor 2 (MT2) antibody neutralisation test. Immunohistochemistry of normal ileal epithelium tissue stained using MT2 antibody ABIN122307 (A). Immunohistochemistry of a consecutive section where the antibody was pre-incubated with the corresponding peptide SP4391CP (B).
(TIF)
