# Supplementary figures and images for: Melatonin Immunoreactivity in Malignant Small Intestinal Neuroendocrine Tumours
Source: PLoS One. 2016 Oct 13;11(10):e0164354. doi: 10.1371/journal.pone.0164354 (PMC5063280; doi:10.1371/journal.pone.0164354)

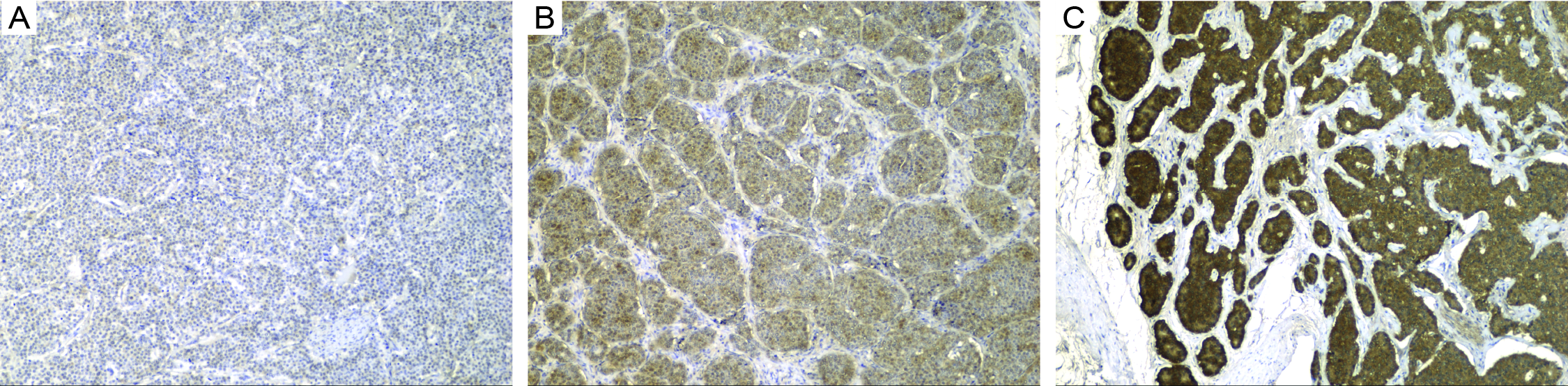

Supplement: S1 Fig — Representative examples of melatonin immunohistochemistry intensities of tumour tissue scored using both manual and computerised methods, weak (A), moderate (B) and strong (C). (TIF) [file pone.0164354.s001.tif]

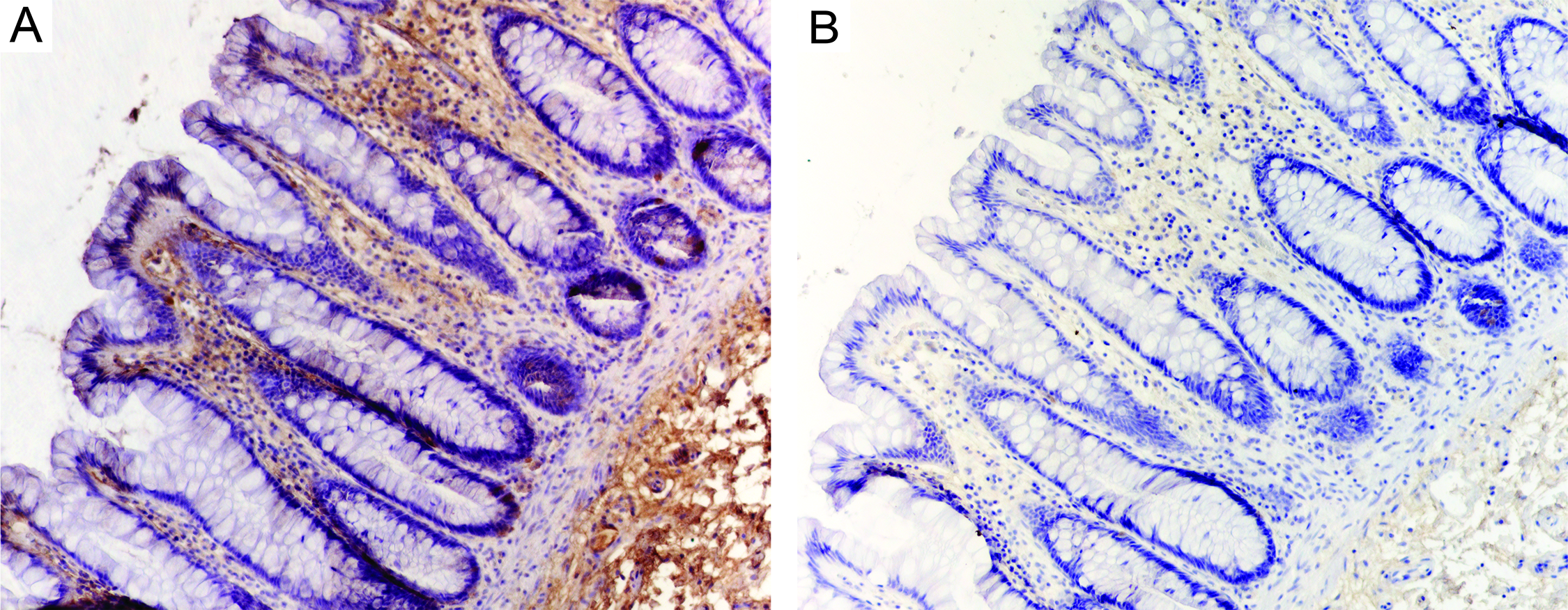

Supplement: S2 Fig — Melatonin immunohistochemistry of normal human rectal epithelium stained using rabbit-anti melatonin antibody 0100–0203 (A). Melatonin immunohistochemistry of a consecutive section where antibody was pre-incubated with 0.1 mg/mL melatonin to ensure antibody specificity (B). (TIF) [file pone.0164354.s002.tif]

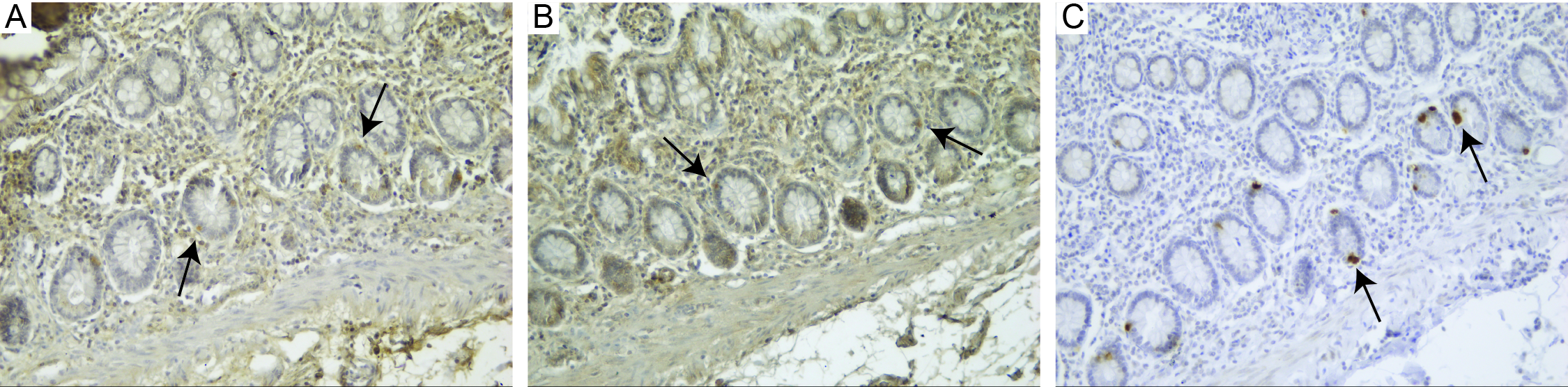

Supplement: S3 Fig — Melatonin immunohistochemistry of normal ileal epithelium stained using rabbit-anti melatonin antibody 0100–0203 (A). Melatonin immunohistochemistry of a consecutive section where antibody was pre-incubated with 0.1 mg/mL serotonin to ensure antibody specificity (B). Serotonin immunohistochemistry of consecutive section stained using anti-serotonin antibody 5HT-H209 (C). Arrowheads indicate enterochomaffin cells. (TIF) [file pone.0164354.s003.tif]

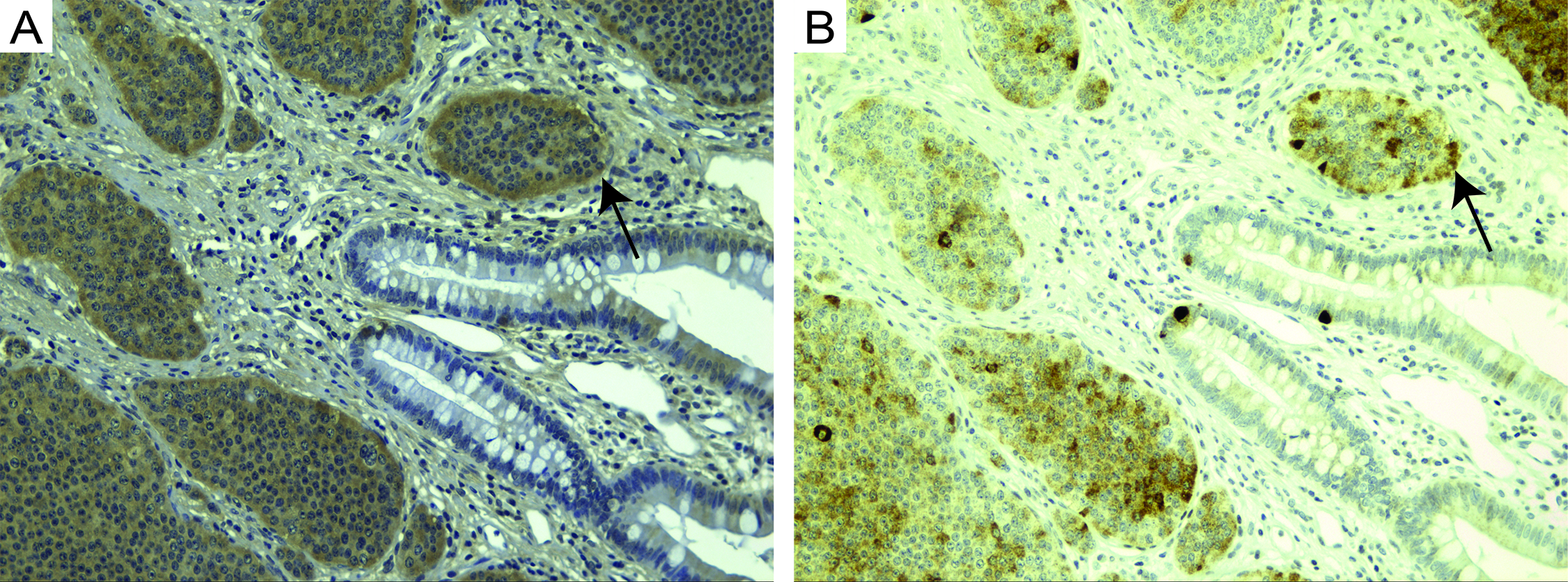

Supplement: S4 Fig — Melatonin immunohistochemistry of small intestinal neuroendocrine tumour stained using rabbit-anti melatonin antibody 0100–0203 (A). Serotonin immunohistochemistry of a consecutive section stained using anti-serotonin antibody 5HT-H209 (B). Arrowheads indicate cluster of tumour cells. (TIF) [file pone.0164354.s004.tif]

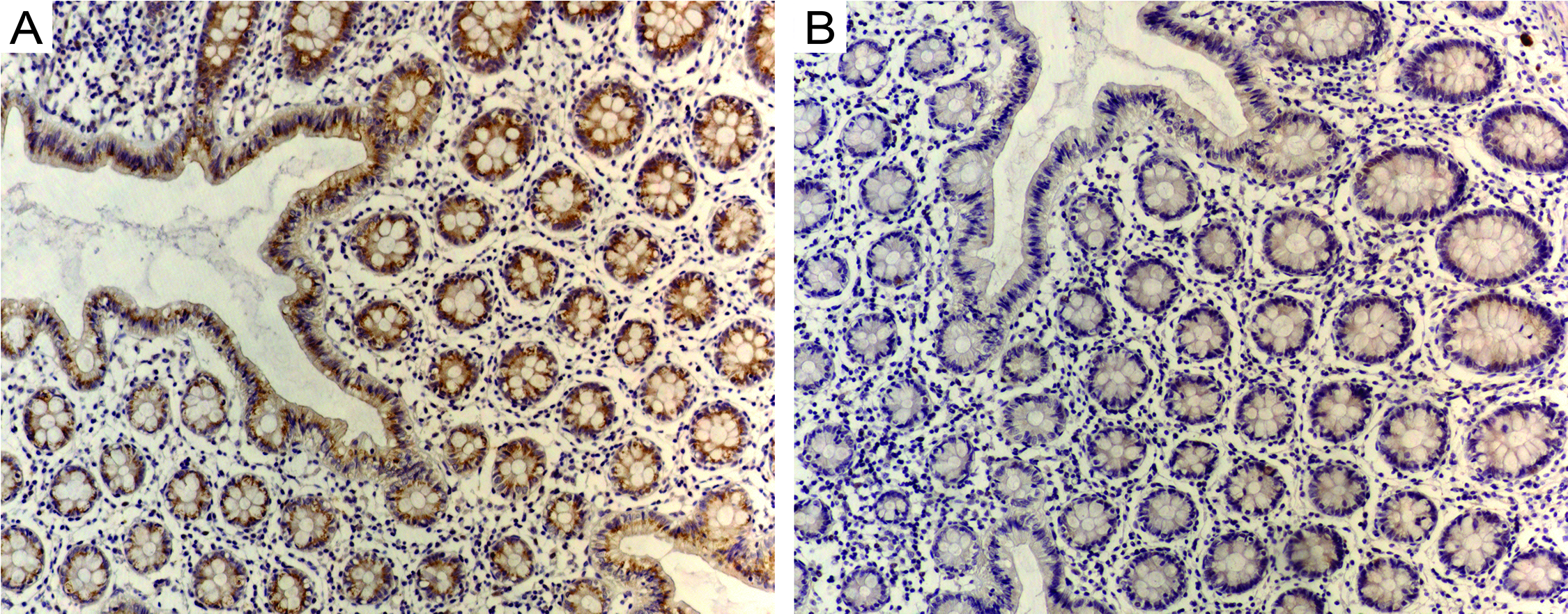

Supplement: S5 Fig — Immunohistochemistry of normal ileal epithelium tissue stained using MT1 antibody sc13179 (A). Immunohistochemistry of a consecutive section where the antibody was pre-incubated with the corresponding peptide sc-13179P (B). (TIF) [file pone.0164354.s005.tif]

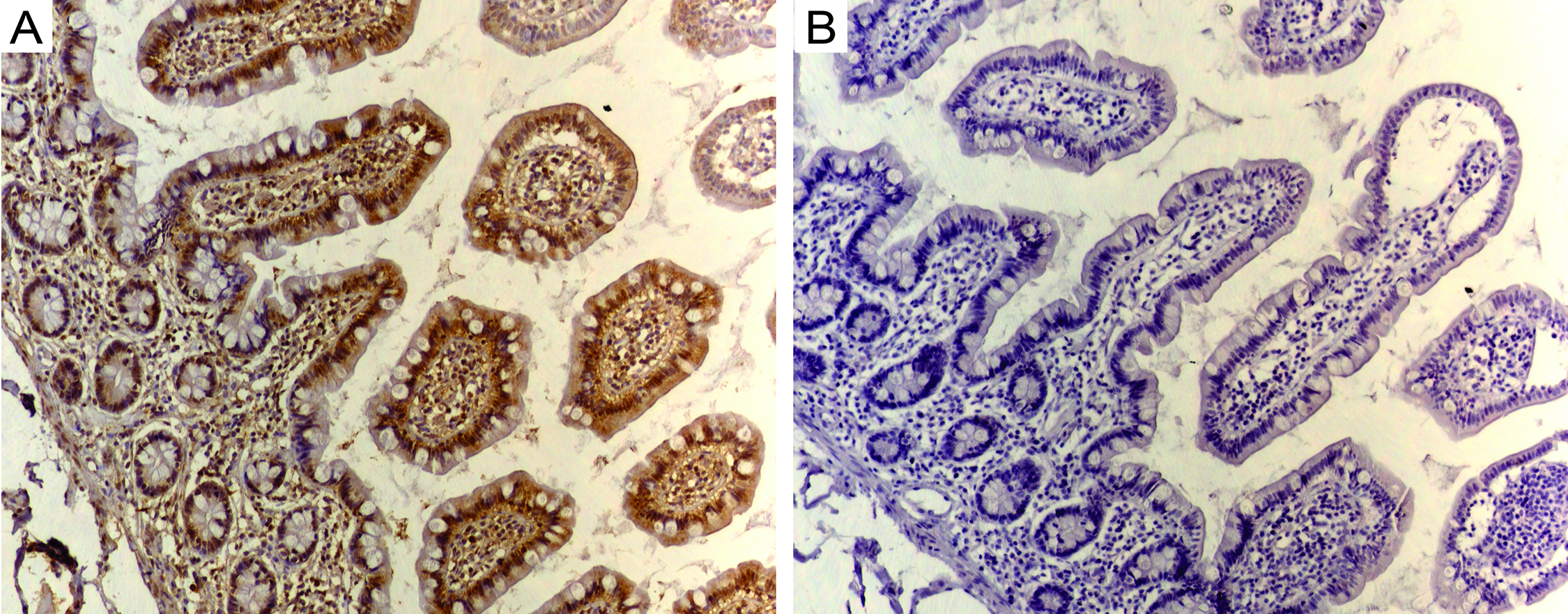

Supplement: S6 Fig — Immunohistochemistry of normal ileal epithelium tissue stained using MT2 antibody ABIN122307 (A). Immunohistochemistry of a consecutive section where the antibody was pre-incubated with the corresponding peptide SP4391CP (B). (TIF) [file pone.0164354.s006.tif]
